# Dose-Dependent Antidepressant-Like Effects of Cannabidiol in Aged Rats

**DOI:** 10.3389/fphar.2022.891842

**Published:** 2022-07-01

**Authors:** Elena Hernández-Hernández, M. Julia García-Fuster

**Affiliations:** ^1^ IUNICS, University of the Balearic Islands, Palma, Spain; ^2^ Health Research Institute of Balearic Islands (IdISBa), Palma, Spain

**Keywords:** aging, late-life depression, antidepressants, rat, forced-swim test, hippocampus, CB receptors, neurogenesis

## Abstract

Aging predisposes to late-life depression and since antidepressants are known to change their efficacy with age, novel treatment options are needed for our increased aged population. In this context, the goal of the present study was to evaluate the potential antidepressant-like effect of cannabidiol in aged rats. For this purpose, 19–21-month-old Sprague–Dawley rats were treated for 7 days with cannabidiol (dose range: 3–30 mg/kg) and scored under the stress of the forced-swim test. Hippocampal cannabinoid receptors and cell proliferation were evaluated as potential molecular markers underlying cannabidiol’s actions. The main results of the present study demonstrated that cannabidiol exerted a dose-dependent antidepressant-like effect in aged rats (U-shaped, effective at the intermediate dose of 10 mg/kg as compared to the other doses tested), without affecting body weight. None of the molecular markers analyzed in the hippocampus were altered by cannabidiol’s treatment. Overall, this study demonstrated a dose-dependent antidepressant-like response for cannabidiol at this age-window (aged rats up to 21 months old) and in line with other studies suggesting a beneficial role for this drug in age-related behavioral deficits.

## Introduction

Aging is the strongest risk factor for most chronic disorders (e.g., Bektas et al., 2018), and in addition to changes in cognitive performance (Rosenzweig and Barnes, 2003; Mattson and Magnus, 2006), some other key features are particularly prone to decline, such as affective-like behavior, predisposing to the development of what is called late-life depression (McKinney and Sibille, 2013). In fact, the prevalence of depression in the population aged over 80 years has been shown to be around 15–20% (Stek et al., 2004), with the corresponding increased prescription of antidepressant drugs (Sonnenberg et al., 2008). Surprisingly, antidepressant-like treatments are known to change their efficacy with age [e.g., reviewed by Felice et al. (2015)]; however, not much research is focused on characterizing classical antidepressant-like responses and/or novel therapeutical options for this age group (see some representative recent preclinical studies: Fernández-Guasti et al., 2017; Mastrodonato et al., 2022), posing a health problem for our continuously increasing elderly population. In the clinic, the only approach taken with elderly patients includes lowering the doses to adjust for a slower metabolism. Remarkably, the described state of increased negative effect emerging with age can be modeled in naïve aging rodents (e.g., from middle-age and on; Hernández-Hernández et al., 2018 and references within; also Herrera-Pérez et al., 2008), providing a preclinical platform in which to test novel antidepressant drugs (e.g., Hernández-Hernández and García-Fuster, 2021).

In the context of characterizing novel treatment options, cannabidiol, a non-psychomimetic phytocannabinoid found in *Cannabis sativa*, has demonstrated a valuable role in ameliorating certain stress-related psychiatric symptoms in rodent models (e.g., Marco et al., 2011; Campos et al., 2016, 2017; Haney and Evins 2016; García-Gutiérrez et al., 2020; Gonzalez-Cuevas et al., 2021; Stanciu et al., 2021), with a great safety potential (Pisani et al., 2021). Interestingly, prior preclinical studies have reported that the antidepressant- and/or anxiolytic-like effects induced by cannabidiol [reviewed recently by García-Gutiérrez et al. (2020) and Melas et al. (2021)] are sex- (Silote et al., 2021; Ledesma-Corvi and García-Fuster, 2022; Martín-Sánchez et al., 2022), stress- (Shbiro et al., 2019; Bis-Humbert et al., 2021a; Ledesma-Corvi and García-Fuster, 2022), and/or age-related (e.g., different efficacy in adolescent vs. adult rats: Bis-Humbert et al., 2020), while studies reporting the potential beneficial effects in older populations are scarce (in addition to its anti-oxidant and anti-inflammatory potential; Dash et al., 2021 and references within). Against this background, the goal of this study was to evaluate whether cannabidiol could exert an antidepressant-like response as measured in the forced-swim test in aged rats.

Several studies have been centered on elucidating cannabidiol’s actions on improving stress-related alterations, involving multiple targets (reviewed by Silote et al., 2019; García-Gutiérrez et al., 2020), such as a multimodal pharmacologic profile over the endocannabinoid system (Bisogno et al., 2001; Thomas et al., 2007; Pertwee, 2008; Campos et al., 2012; Laprairie et al., 2015; Martínez-Pinilla et al., 2017; Tham et al., 2019), an agonistic potential over TRPV1 receptors (Bisogno et al., 2001), as well as the regulation of other neurotransmitter systems [i.e., serotoninergic, opioidergic, and dopaminergic; reviewed in Silote et al. (2019)] or neuroprotective targets (i.e., hippocampal neurogenesis; Marchalant et al., 2009; Fogaça et al., 2018; Luján et al., 2018, 2020). For that reason, and since hippocampal function is altered with aging (Rosenzweig and Barnes, 2003), the current study evaluated the potential regulation of CB1 and CB2 receptors, as well as that of an early stage of neurogenesis (i.e., cell proliferation) following cannabidiol’s treatment in the hippocampus of aged rats.

## Methods

### Animals

**FIGURE 1:**
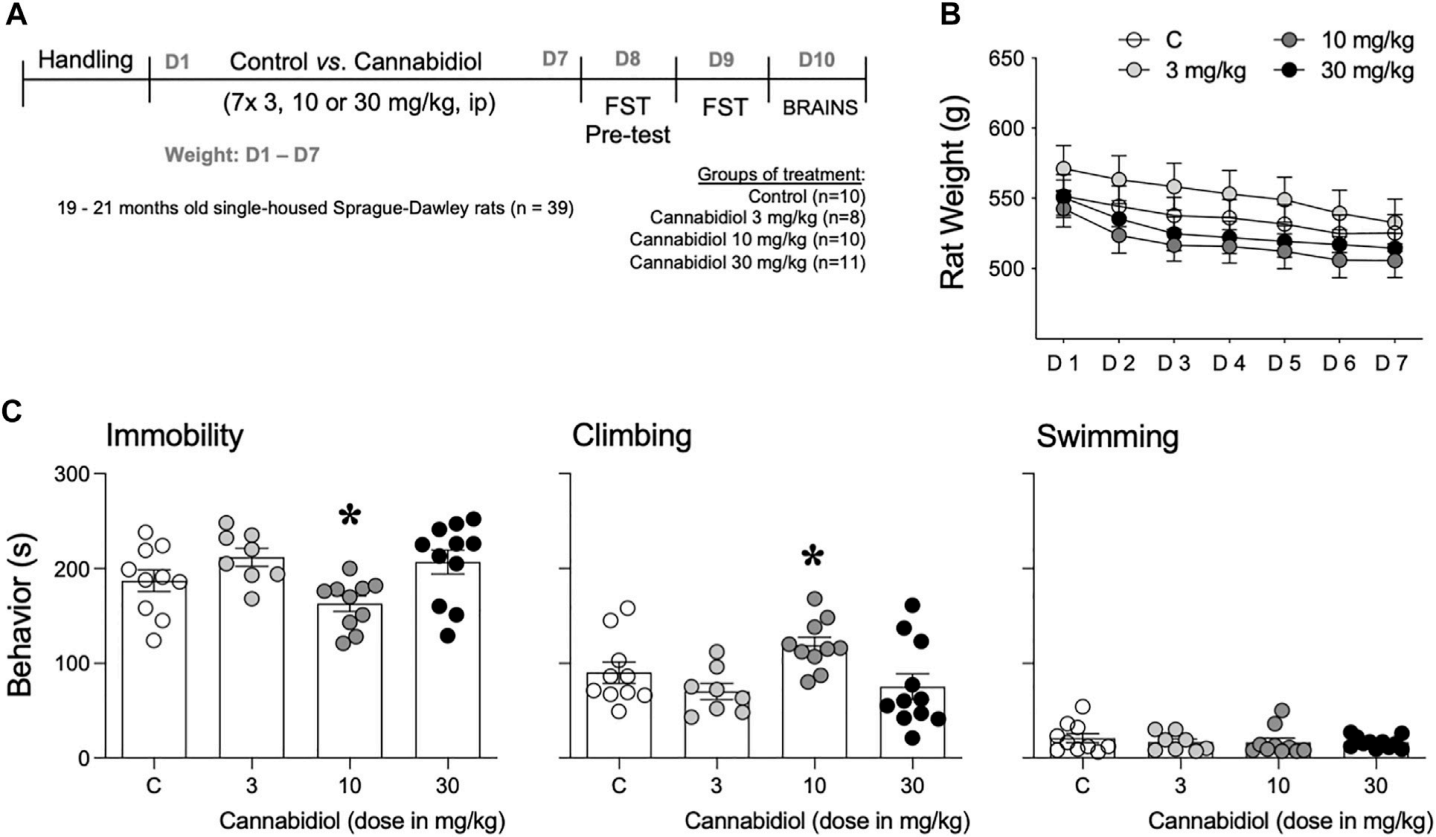
**(A)** Experimental timeline. **(B)** Monitorization of body weight (g) during the experimental treatment. Data represent mean ± SEM of the body weight (g). A two-way repeated-measures ANOVA did not detect a significant effect of treatment. **(C)** Antidepressant-like effect as measured in the forced-swim test. Data represent mean ± SEM of the time spent (s) immobile, climbing, or swimming. Individual values are shown for each rat (symbols). One-way ANOVAs detected significant changes for immobility and climbing. Multiple comparisons were performed with Tukey’s test: *p < 0.05 vs. the other doses tested, both 3 and 30 mg/kg of cannabidiol. Groups of treatment: control (C, *n* = 10) and cannabidiol (3 mg/kg, *n* = 8; 10 mg/kg, *n* = 10; and 30 mg/kg, *n* = 11).

For this study, 39 male Sprague–Dawley rats (bred in the animal facility at the University of the Balearic Islands) were used when they were 19–21 months old (Figure 1A). The rats were housed under standard vivarium conditions (22°C, 70% humidity, and 12-h light/dark cycle, lights on at 8:00 a.m.) with *ad libitum* access to a standard diet and tap water. Following size requirements (animals housed per standard cage), the rats were individually housed for several months before testing started. All procedures were performed during the light period (between 8:00 h and 15:00 h), complied with ARRIVE guidelines (Percie du Sert et al., 2020), EU Directive 2010/63/EU for animal experiments, and Spanish Royal Decree 53/2013, and were approved by the Local Bioethical Committee and the Regional Government. All efforts were made to minimize the number of rats used and their suffering.

### Repeated Cannabidiol Treatment

All rats were handled for 5 days prior to drug treatment, during which they were separated into four groups and received either a daily dose of cannabidiol (3, 10, or 30 mg/kg, i.p.; *n* = 8, 10, and 11, respectively) (purity ≥98%; THC Pharm, Germany) or vehicle (1 ml/kg of DMSO, control group; *n* = 10) for 7 days. The doses of cannabidiol were selected based on a prior study from our research group showing age- and dose-dependent antidepressant-like responses (i.e., different dose-efficacy during adolescence vs. adulthood in male naïve rats; Bis-Humbert et al., 2020). Body weight was daily monitored through the treatment process (D1–D7).

### Forced-Swim Test

To ascertain cannabidiol’s antidepressant-like response in aged rats we used the forced-swim test, since it is a standardized test for screening antidepressant-like responses under a stressful situation (Slattery and Cryan, 2012). For this purpose, and following prior well-established procedures in our group (Bis-Humbert et al., 2020; 2021b), rats were individually exposed to a 15 min pre-test on D8, and a 5-min test on D9 (Figure 1A) that was videotaped. The water tanks (41 cm high × 32 cm diameter) were filled with water up to 25 cm depth (25 ± 1°C) and were changed for each rat. Videos were coded and blindly analyzed using Behavioral Tracker Software (CA, United States) to calculate the time spent (s) immobile or active (climbing or swimming).

### Hippocampal Neurochemical Correlation

All rats were sacrificed by rapid decapitation on D10 (Figure 1A), their brains were extracted, and both hemispheres were separated. On the one hand, the right hippocampus was freshly dissected and frozen in liquid nitrogen until the contents of CB receptors were evaluated in total homogenates (40 μg) by Western blot analysis with anti-CB1 (1:2000; Abcam, Cat. No. 23703, United Kingdom) or anti-CB2 (1:1000; Cayman Chemical, Cat. No. 101550, United States) primary antibodies, as previously detailed by our group (García-Cabrerizo and García-Fuster, 2016; Bis-Humbert et al., 2021a). Low quantities of total homogenates (15 μg) were loaded to detect β-actin (1:10000; Sigma-Aldrich, clone AC-15, United States), which was used as a negative loading control, since its content was not altered by any of the treatment conditions. Each sample was run at least three times in different gels, and percent changes were calculated for each rat as compared to control-treated samples loaded in the same gels. On the other hand, the left hemisphere was quickly frozen and stored until the entire hippocampus (−1.72 to −6.80 mm from Bregma) was cryostat-cut (30 μm sections) and slide mounted (8 sections/slide, 8 slides/series, and 3 series/animal from the most anterior to the posterior part of the hippocampus) to evaluate the rate of cell proliferation with the anti-Ki-67 antibody (1:40,000; kindly provided by Dr. Huda Akil and Dr. Stanley J. Watson, University of Michigan, United States) by immunohistochemistry as detailed earlier (García-Fuster et al., 2010; García-Cabrerizo et al., 2015; García-Fuster et al., 2017). The number of immunostained Ki-67 + cells was quantified using a Leica DMR light microscope (63× objective lens) in all sections by a blind experimenter to the treatment groups and as previously described in detail (e.g., García-Fuster et al., 2010, 2011, 2017).

### Statistics

All data were analyzed with GraphPad Prism, Version 9.3.1 (GraphPad Software, United States). The results are expressed as mean values ± standard error of the mean (SEM), with individual symbols for each rat shown within bar graphs. The changes in body weight were analyzed with two-way repeated-measures ANOVA. Potential overall changes in behavior (s) in the forced-swim test (i.e., immobility, climbing, or swimming) or in the content of brain markers (i.e., CB receptors and Ki-67 + cells) were evaluated by one-way ANOVAs followed by Tukey’s multiple comparisons test. The level of significance was set at *p* ≤ 0.05.

## Results

### Cannabidiol Did Not Alter Normal Body Weight (g) in Aged Rats

Although no changes were observed by cannabidiol’s treatment (F_3,35_ = 1.18, *p* = 0.329), there was a significant effect of day (F_6,210_ = 68.15, *p* < 0.001), probably driven by the observed moderate decreases in body weight for all groups during the course of the experimental procedure, and most probably caused by the stress of cannabidiol’s treatment (Figure 1B).

### Dose-Dependent Antidepressant-Like Effects of Cannabidiol in Aged Rats

When evaluating the antidepressant-like potential of cannabidiol (3, 10, and 30 mg/kg; i.p.) in the forced-swim test, although a one-way ANOVA detected a significant difference in the time-aged rats spent immobile (F_3,35_ = 4.18, *p* = 0.012; Figure 1C), this effect was mainly driven by the dose of 10 mg/kg, which significantly reduced immobility as compared to the other doses tested (−49 ± 16 s, ∗*p* = 0.021 vs. 3 mg/kg; −44 ± 15 s, ∗*p* = 0.026 vs. 30 mg/kg; U-shaped dose response; Figure 1C). Interestingly, the decrease observed in immobility paralleled an increase in the time rats spent climbing (F_3,35_ = 3.89, *p* = 0.017), with significant changes when comparing the dose of 10 mg/kg vs. 3 mg/kg (+49 ± 16 s, ∗*p* = 0.026) and 30 mg/kg (+44 ± 15 s, ∗*p* = 0.031; Figure 1C). Finally, no changes were observed in swimming behavior (F_3,35_ = 0.33, *p* = 0.803).

### Cannabidiol Did Not Modulate CB1 and CB2 Receptors or the Rate of Early Cell Proliferation in the Hippocampus

**FIGURE 2:**
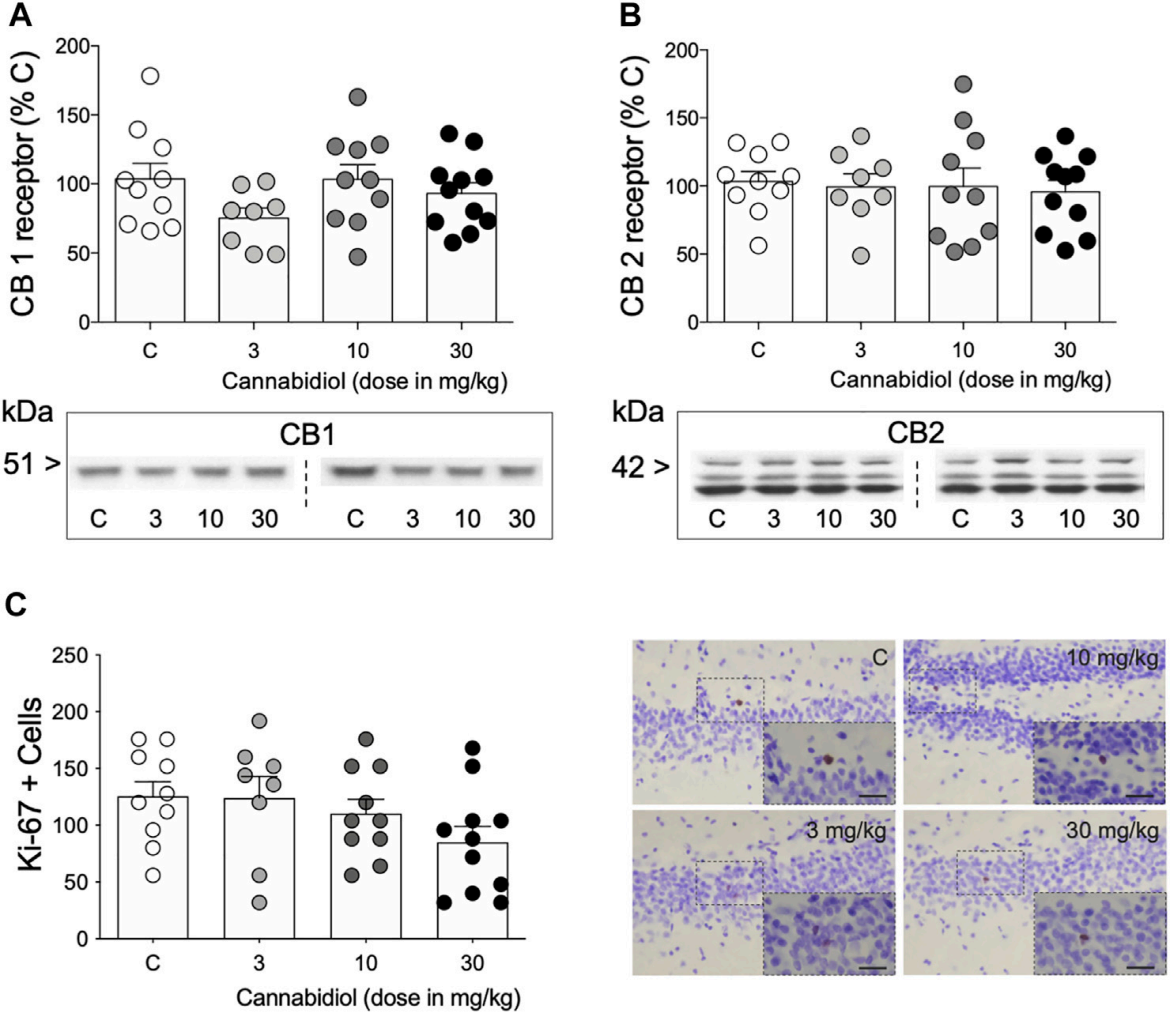
Modulation of hippocampal molecular markers by repeated cannabidiol treatment. **(A)** CB1 and **(B)** CB2 receptors as measured by Western blot analysis. Data represent mean ± SEM of CB1 and CB2 protein contents expressed as % change vs. control-treated rats. Individual values are shown for each rat (symbols). One-way ANOVAs did not detect any significant changes. Representative immunoblots are shown depicting CB1 and CB2 labeling. **(C)** Ki-67 + cells in the dentate gyrus as measured by immunohistochemistry analysis. Data represent mean ± SEM of Ki-67 + cells. Individual values are shown for each rat (symbols). A one-way ANOVA did not detect any significant changes. Representative images of Ki-67 + cells (brown labeling in the blue granular layer) were taken using a light microscope and quantified using a 63× objective lens. Scale bar: 30 μm.

When evaluating some of the potential molecular markers regulated by cannabidiol and that could parallel its behavioral actions, the results showed no changes in CB1 (F_3,35_ = 1.68, *p* = 0.189; Figure 2A) and CB2 (F_3,35_ = 0.11, *p* = 0.105; Figure 2B) receptors, nor in the rate of cell proliferation (Ki-67 + cells: F_3,35_ = 1.78, *p* = 0.168; Figure 2C) in the hippocampus of aged rats as measured 3 days post-treatment.

## Discussion

The main results of the present study demonstrated that cannabidiol is capable of exerting a dose-dependent antidepressant-like response in aged rats, without inducing changes in body weight. None of the molecular markers analyzed in the hippocampus (CB receptors and cell proliferation) were altered by cannabidiol treatment.

Cannabidiol induced a dose-dependent antidepressant-like effect in aged rats, as observed in the forced-swim test by a decrease in immobility paired with an increase in climbing. Interestingly, these effects were dose-dependent, being efficacious only with the intermediate dose tested (10 mg/kg) and as compared to lower (3 mg/kg) or higher (30 mg/kg) doses. In regards to the effective dose-range for cannabidiol’s antidepressant-like efficacy through its ability to reduce immobility scores under the stress of a forced-swim test, prior studies suggested that lower doses were needed for mice (3–30 mg/kg) than rats (30–60 mg/kg) as reviewed in Silote et al. (2019). However, a recent study compared cannabidiol’s antidepressant-like potential in adolescent vs. adult rats and demonstrated that while a dose of 30 mg/kg was needed in adulthood to induce changes in the forced-swim test, only the intermediate dose tested (10 mg/kg) rendered efficacious in adolescent rats (Bis-Humbert et al., 2020), demonstrating a similar U-shaped dose–response curve for adolescent and aged rats, and lowering the dose at which cannabidiol could induce an antidepressant-like effect in older rats. A similar U-shaped pattern has been previously reported for cannabidiol’s anxiolytic-like effects, with effective responses at an intermediate dose, but no change with lower or higher doses (García-Gutiérrez et al., 2020 and references within) and for cannabidiol’s medicinal usage for cocaine-related behaviors has been reported (Nedelescu et al., 2022). Interestingly, the reduced rate in immobility was paralleled by an increase in climbing behavior, as previously reported for cannabidiol (Bis-Humbert et al., 2020; Ledesma-Corvi and García-Fuster, 2022) and in line with the antidepressants that exert their actions through the modulation of the noradrenergic system (Detke et al., 1995). In conjunction, and to the best of our knowledge, this is the first study to demonstrate that cannabidiol is capable of inducing dose-dependent responses in the forced-swim test indicative of an antidepressant-like response in naïve aged rats, which physiologically show increased negative effect as compared to younger rats (Hernández-Hernández et al., 2018). These data provide a new therapeutical option for late-life depression that deserves further characterization. In this context, the observed lack of effect of cannabidiol on body weight at the doses tested suggested a good safety profile for cannabidiol in aged rats. Although some prior studies reported that repeated cannabidiol treatment could decrease normal body weight gain in adult rats (Ignatowska-Jankowska et al., 2011; Santiago et al., 2019), an effect probably mediated by CB2 receptors (Ignatowska-Jankowska et al., 2011), these effects seemed to be age dependent, since they replicated in a separate study for adult rats, but were not observed when cannabidiol was administered at the same doses during adolescence (Bis-Humbert et al., 2020) or in aged rats (present study). Moreover, one might speculate that the effects observed in the forced-swim test could be driven by changes in locomotor activity; however, several studies with doses up to 60 mg/kg of cannabidiol reported no changes in spontaneous locomotion when administered alone (e.g., Taffe et al., 2015; Anooshe et al., 2021; Ledesma et al., 2021), including our own evaluations when measuring distance traveled in the open field test (e.g., Bis-Humbert et al., 2020; Ledesma-Corvi and García-Fuster, 2022).

In an attempt to study some of the potential molecular targets and/or markers modulated by cannabidiol in aged rats, we explored the regulation of CB receptors (e.g., Fogaça et al., 2018) and a marker of an early stage of hippocampal neurogenesis (i.e., cell proliferation; Luján et al., 2018, 2020; Bis-Humbert et al., 2020). The results showed no changes in any of the markers analyzed. As for CB receptors, cannabidiol did not alter the protein content of CB receptors in the hippocampus at the time rats were sacrificed. Further studies should be carried out to evaluate the dynamics on how cannabidiol modulates CB receptors and include other brain regions. Also, other age-related changes such as the observed decrease in cannabinoid receptor binding and mRNA levels in aged rats (Romero et al., 1998) must be considered. Moreover, regarding cell proliferation, our prior study also showed a lack of regulation by cannabidiol in adolescent or adult rats at the same doses tested here and at the same specific time-points of analysis (Bis-Humbert et al., 2020), reinforcing the idea that the previously described beneficial effects of cannabidiol on improving cell genesis were observed in the context of prior exposure to a given stressor (Luján et al., 2018, 2020). In any case, these results are limited by the fact that brains were analyzed 3 days post-treatment (1 day after the observed antidepressant-like effect), with this timing being a particular photo-finish, and not necessarily correlative of the behavioral effects, and therefore conclusions should be made cautiously and in the context of this limitation. Further studies should collect brains at the specific time when the antidepressant-like effect was observed.

Ideally, we would have included female rats to compare cannabidiol’s effects at this age range, since depression is about twice as common in women as in men (e.g., Labaka et al., 2018), and preclinical studies evaluating antidepressant-like responses in females are scarce in general, but even more so for female aged rodents (reviewed in Felice et al., 2015; Fernández-Guasti et al., 2017). Unfortunately, no female rats were available at the time of our experiments, and therefore, the effect of sex as a biological variable could not be included in the present study. In any case, prior studies suggested certain inefficacy for cannabidiol’s antidepressant-like potential in female adult rats (e.g., Silote et al., 2021), including our own (Ledesma-Corvi and García-Fuster, 2022), and thus recommending future studies to better characterize the potential of this drug in both sexes and at all age-ranges.

In conclusion, this study increased the age-window at which cannabidiol exerted dose-dependent responses in this behavioral test, to include aged rats (up to 21 months old), at which it could be considered as a potential antidepressant, and in line with other studies suggesting a beneficial role for this drug in age-related behavioral deficits. Further experiments should evaluate other parameters (i.e., a wider range of doses, longer treatment paradigms, and models of induced depression) and include female aged rats to not only characterize the behavioral potential of cannabidiol but also try to better understand the molecular mechanisms underlying its actions.

## Data Availability

The orginal contributions presented in this study are included in the article; further inquires can be directed to the corresponding author.
